# Editorial: Roles of flavonoids in crop quality improvement and response to stresses

**DOI:** 10.3389/fpls.2023.1210666

**Published:** 2023-05-26

**Authors:** Quan Zhang, Sunil S. Gangurde, Xinlei Yang, Chuanzhi Zhao

**Affiliations:** ^1^ Shandong Provincial Key Laboratory of Plant Stress Research, College of Life Sciences, Shandong Normal University, Jinan, China; ^2^ Crop Protection and Management Research Unit, United States Department of Agriculture-Agricultural Research Service (USDA-ARS), Tifton, GA, United States; ^3^ Department of Plant Pathology, University of Georgia, Tifton, GA, United States; ^4^ College of Agronomy, Hebei Agricultural University, Baoding, China; ^5^ Institute of Crop Germplasm Resources (Institute of Biotechnology), Shandong Academy of Agricultural Sciences, Jinan, China; ^6^ Shandong Provincial Key Laboratory of Crop Genetic Improvement, Ecology and Physiology, Jinan, China

**Keywords:** flavonoids biosynthesis, gene identification, biotic and abiotic stress tolerance, crop breeding evaluation, flavonoid biosynthesis engineering

## Introduction

Flavonoids play a variety of biological functions in plants. More than anthocyanins pigments displaying red, blue and purple, in flowers, fruits and leaves, which determine economic traits of crops and ornamental plants, they play multiple functional roles in plant-environment interactions which protect plants from biotic and abiotic stresses (Zhang et al., 2014; Landi et al., 2015; Qi et al., 2020). Importantly, flavonoids have critical functions in antioxidant and antitumor, as well as promoting blood circulation, thus it is benificial to health of human from eating edible seeds and fruits rich in flavonoids (Zhou et al., 2004; Li et al., 2018). Here, this Research Topic focus on progress on the function and role of flavonoids in plants encountering abiotic stresses, and the genetic engineering for manipulation of the flavonoids biosynthesis pathways to enhance flavonoid content and the abiotic stress tolerance in plants, especially crops.

## Interaction between flavonoids of plants and their environments

Wine grapes are often grown in regions characterized by dry and warm summers, and a proper irrigation strategy is essential for obtaining high-quality berries and wines (Buesa et al., 2021; Palai et al., 2022; Romero et al., 2022). Palai et al. studied that effect of the regulated deficit irrigation (RDI) on berry flavonoid content and its related biosynthetic pathways in Sangiovese grapevines, including the moderate (RDI-1M) or severe (RDI-1S) water deficit from pea-size berry to veraison, moderate (RDI-2M) or severe (RDI-2S) water deficit from veraison through harvest, and severe during the lag-phase (RDI-LS). They found a highest accumulation of anthocyanin in berries from both RDI-1 treatments, and expression of many genes in the flavonoid pathway was upregulated from beginning of veraison until harvest. Both post-veraison water deficit also increased anthocyanin concentration, but to a lesser degree than RDI-1. Particularly, the moderate deficit irrigation regardless of the applied periods in pre- or post-veraison, enhanced anthocyanin accumulation compared with that in severe water-deficit. Flavonol concentration was higher in RDI-1S berries, due to the upregulated expression of genes encoding flavonol synthases and the flavonol-3-O-glycosyltransferases. Overall, the study highlighted that the timing and intensity of deficit irrigation can modulate berry flavonoid accumulation as well as phenylpropanoid/flavonoid pathways, suggesting a proper management of deficit irrigation is critical for berry to enhance accumulation of anthocyanin and/or flavonol.

Alkali stress is highly destructive to the ecology, seriously affecting the soil structure and the growth of plants (Fancy et al., 2017). *Nitraria tangutorum* had strong alkaline resistance, accompanying a higher accumulation of flavonoid and anthocyanin during the period. Zhang et al. examined the role of the exogenous application of ABA and sodium nitroprusside (SNP) on defensive response of *N. tangutorum* to alkaline stress. They found that exogenous ABA and SNP significantly increased the plant height, fresh weight, relative water content and degree of succulency, as well as reduced the growth inhibition and physiological damage caused by alkali stress in *N. tangutorum* seedlings. However, SNP has a better effect on the improvement of photosynthetic efficiency and regulation of carbohydrate accumulation, while ABA has a more obvious effect on regulating flavonoid accumulation. Alkali stress also induced accumulation of endogenous NO and ABA, which might play a positive regulated role on defensive response of *N. tangutorum* to alkaline stress.

Powdery mildew is a fungal disease devastating to wheat, causing significant loss in quality and yield (Fofana et al., 2005; Bajpai et al., 2019). Xu et al. conducted the combined analysis of transcriptome and metabolome in susceptible Jimai229 wheat and resistant HHG46 with and without powdery mildew inoculation. They revealed that the flavone and flavonol biosynthesis pathways were significantly enriched in both cultivars following powdery mildew inoculation, which was consistent with the upregulated flavonoid biosynthesis genes and increased accumulation of total flavonoid. Moreover, exogenous flavonoid treatment of inoculated plants led to fewer and smaller powdery mildew spots on the wheat leaves. Thus flavonoids is suggested to confer resistance to powdery mildew in wheat.

## Identification of function of candidate genes/loci involved in flavonoid accumulation, growth and development of plants

Flavonol synthases play important role in regulating flavonoid metabolism, specifically the flavonol and anthocyanin branching pathways (Vu et al., 2015). Guo et al. used the targeted LC–MS to determine flavonoid-related substances in overexpression of *GbFLSa* in *Populus* poplar and CK, and revealed the content of proanthocyanins including catechin, epicatechin, epigallocatechin and gallocatechin, as well as expression levels of two *DFRs*, three *ANSs* and two *LARs* in transgenic poplars were significantly lower than that in nontransgenic plants. The study indicates that *GbFLSa* overexpression plays a negative regulatory role in biosynthesis of proanthocyanins.

Peanut (*Arachis hypogaea*), with variegated testa color representing a distinct regulation pattern of anthocyanin accumulation in integument cells, has attractive appearance and higher market value. Chen et al. constructed two populations from the crosses between Fuhua 8 (pure-pink testa) and Wucai (red on white variegated testa), Quanhonghua 1 (pure-red testa) and Wucai, respectively, and identified the genetic locus underlying variegated testa color in peanut. They revealed that the pigmentation of colored region in red on white variegated testa was controlled by a previous reported gene *AhRt1*, while the formation of white region in variegated testa was controlled by genetic locus *AhVt1* (*Arachis hypogaea Variegated Testa 1*). The molecular markers closely linked to the *AhVt1* were developed, and the marker-assisted selection was used to develop new variegated testa peanut lines. The findings accelerate the breeding program for developing new peanut varieties with “colorful” testa colors, and laid a foundation for map-based cloning of genes responsible for variegated testa.

Ginkgo possessing its distinctive branching and fan-shaped leaves, has become one of the world’s most popular ornamental street trees (Crane, 2019). Flavonoids have been associated with the regulation of auxin transport (Santelia et al., 2008). Ni et al. investigated the phenotypic changes in transgenic tobacco (*Nicotiana tabacum*) overexpressing ginkgo *GbDFR6*. Pleiotropic defects in root and leaf development indicated an impaired auxin transport in transgenic tobacco plants, and eight flavonoids identified are ideal candidates as novel regulators of auxin transport by UPLC-ESI-MS/MS analysis. Delayed flowering in transgenic tobacco plants indicated anthocyanin or flavonoid is involved in the regulation of flowering time, which provide a valuable cue to produce the late flowering tobacco variety. The research suggested the novel and multiple roles of *GbDFR6* in ginkgo.

## Author contributions

QZ and CZ drafted the manuscript. SG and XY provided input and comments to the draft. All authors listed have made a substantial, direct, and intellectual contribution to the work and approved it for publication.

## References

[B1] Bajpai S. Shukla P. S. Asiedu S. Pruski K. Prithiviraj B. (2019). A biostimulant preparation of brown seaweed *Ascophyllum nodosum* suppresses powdery mildew of strawberry. Plant Pathol. J. 35 (5), 406–416. doi: 10.5423/PPJ.OA.03.2019.0066 31632216 PMC6788409

[B2] Buesa I. Intrigliolo D. S. Castel J. R. Vilanova M. (2021). Influence of water regime on grape aromatic composition of Muscat of Alexandria in a semiarid climate. Scientia Horticulturae. 290 (2), 110525. doi: 10.1016/j.scienta.2021.110525

[B3] Crane P. R. (2019). An evolutionary and cultural biography of ginkgo. *Plants*, *People* . Planet 1 (1), 32–37. doi: 10.1002/ppp3.7

[B4] Fancy N. N. Bahlmann A. K. Loake G. J. (2017). Nitric oxide function in plant abiotic stress. Plant Cell Environ. 40 (4), 462–472. doi: 10.1111/pce.12707 26754426

[B5] Fofana B. Benhamou N. McNally D. J. Labbé C. Séguin A. Bélanger R. R. (2005). Suppression of induced resistance in cucumber through disruption of the flavonoid pathway. Phytopathology 95 (1), 114–123. doi: 10.1094/PHYTO-95-0114 18943844

[B6] Landi M. Tattini M. Gould K. S. (2015). Multiple functional roles of anthocyanins in plant-environment interactions. Environ. Exp. Botany. 119, 4–17. doi: 10.1016/j.envexpbot.2015.05.012

[B7] Li H. Sun X. Yu F. Xu L. Miu J. Xiao P. (2018). In silico investigation of the pharmacological mechanisms of beneficial effects of *Ginkgo biloba* l. @ on alzheimer’s disease. Nutrients 10 (5), 589. doi: 10.3390/nu10050589 29747475 PMC5986469

[B8] Palai G. Caruso G. Gucci R. D’Onofrio C. (2022). Deficit irrigation differently affects aroma composition in berries of *Vitis vinifera* l. (cvs sangiovese and merlot) grafted on two rootstocks. Aust. J. Grape Wine Res. 28 (4), 590–606. doi: 10.1111/ajgw.12562

[B9] Qi Y. Gu C. Wang X. Gao S. Li C. Zhao C.. (2020). Identification of the *Eutrema salsugineum* EsMYB90 gene important for anthocyanin biosynthesis. BMC Plant Biol. 20 (1), 186. doi: 10.1186/s12870-020-02391-7 32345216 PMC7189703

[B10] Romero P. Navarro J. M. Ordaz P. B. (2022). Towards a sustainable viticulture: the combination of deficit irrigation strategies and agroecological practices in Mediterranean vineyards. a review and update. Agric. Water Management. 259, 107216. doi: 10.1016/j.agwat.2021.107216

[B11] Santelia D. Henrichs S. Vincenzetti V. Sauer M. Bigler L. Klein M.. (2008). Flavonoids redirect PIN-mediated polar auxin fluxes during root gravitropic responses. J. Biol. Chem. 283 (45), 31218–31226. doi: 10.1074/jbc.M710122200 18718912 PMC2662185

[B12] Vu T. T. Jeong C. Y. Nguyen H. N. Lee D. Lee S. A. Kim J. H.. (2015). Characterization of *Brassica napus* flavonol synthase involved in flavonol biosynthesis in *Brassica napus* l. J. Agric. Food Chem. 63 (35), 7819–7829. doi: 10.1021/acs.jafc.5b02994 26264830

[B13] Zhang Y. Butelli E. Martin C. (2014). Engineering anthocyanin biosynthesis in plants. Curr. Opin. Plant Biol. 19, 81–90. doi: 10.1016/j.pbi.2014.05.011 24907528

[B14] Zhou W. Chai H. Lin P. H. Lumsden A. B. Yao Q. Chen C. (2004). Clinical use and molecular mechanisms of action of extract of *Ginkgo biloba* leaves in cardiovascular diseases. Cardiovasc. Drug Rev. 22 (4), 309–319. doi: 10.1111/j.1527-3466.2004.tb00148.x 15592576

